# The Proclivity of Women to Cancerous Diseases, and to Certain Benign Tumours

**Published:** 1892-06

**Authors:** 


*The Proclivity of Women to Cancerous Diseases, and to Certain Benign Tumours.* By HERBERT SNOW, M.D. (Lond.). Pp. 58. London: J. & A. CHURCHILL. 1891.

We are informed by the title page that this is the substance of a lecture delivered at the Cancer Hospital, on February 6th, 1891; but after carefully reading it, we cannot discover in what way it advances our knowledge of the etiology of cancer. The author's opinion seems to be, that whisky-drinking and smoking are the principal causes of cancer in the male, and that constipation, tight lacing, tea-drinking, and overpressure at school have very much to do with the same result in the female. In his view, all the phenomena of cancer-development are phenomena of cell growth, and all questions of causation resolve themselves into the single one of what stimulates or irritates the cell-elements in a particular area. He considers that the frequency of cancer of the uterus is due to menstruation, "that proud privilege of woman, due to the assumption, by her kind, of the erect position."

We are afraid that we have yet more to learn than the author can teach us, before we can understand anything of the causation of cancer.

